# Making MOOCs meaningful and locally relevant? Investigating IDCourserians—an independent, collaborative, community hub in Indonesia

**DOI:** 10.1186/s41039-016-0032-6

**Published:** 2016-05-04

**Authors:** Manda Firmansyah, Sue Timmis

**Affiliations:** Graduate School of Education, University of Bristol, 35 Berkeley Square, Bristol, BS8 1JA UK

**Keywords:** MOOCs, Learning community, Communities of practice, Collaborative learning, Globalisation, Self-regulated learning

## Abstract

Along with massive open online course (MOOC) budding popularity, some problems have begun to surface. One that appears prominent concerns sustainability: for example, high dropout and low completion rates, which are reported to be less than 10 % on average. In response to growing concerns over these issues, some MOOC providers have begun to campaign for the development of offline supporting communities. Meanwhile in Indonesia, where provider supported learning communities are not yet present, some MOOC learners took the initiative to form their own community, which they called IDCourserians. This paper reports on a qualitative study which aimed to investigate and to make sense what was actually happening in the IDCourserians as an independent MOOC learning community. Nine overarching themes were identified from the collected data that illuminate the purpose of the IDCourserians, the way the community’s members learn MOOCs, and the benefit the participants perceived by joining the IDCourserians. These findings then are discussed further with regard to two key theoretical perspectives: collaborative learning and communities of practice. The paper concludes that where English is not the main spoken language and the hub model is not yet provided, creating face-to-face communities in local communities should be considered, as this may be a better way for learners to experience MOOCs and make their learning more meaningful in the local context. This also offers potential for overcoming the common difficulties in MOOCs, such as a lack of motivation and difficulties in interpreting the material through developing collaborative support activities within a community of practice.

## Introduction

This paper focuses on a study which investigated a learning community based in Jakarta, Indonesia, called IDCourserians, which subscribes to Coursera, one of the massive open online course (MOOC) providers. A MOOC can be understood as a distance education model which is ‘massive’ because it affords thousands or perhaps millions of participants, ‘open’ because anybody can enrol regardless of their background and ‘online’ because all courses are delivered through the Internet using e-learning platforms (Pomerol et al. 2015).

The number of Internet users in Indonesia in 2012 reached about 63 million users and 139 million users in 2015 so that the country is well placed in theory to capitalise on online and distance learning opportunities (Utomo & Rosmansyah 2014). At the time this study was being conducted (2014), MOOCs were still a relatively new educational practice in Indonesia. Though some scholars suggest MOOCs might complement existing higher education institutional practices (Utomo & Rosmansyah 2014), there appears to have been limited development of MOOCs in higher education institutions or associated organisations in Indonesia. There are some reasons for this in terms of bandwidth and connectivity issues (Hollands & Tirthali 2014). However, local development of MOOCs is likely also to be hindered by the dominance of ‘global-institutional spaces’, particularly those from the USA, Canada, Australia, and Western Europe which dominate the market in MOOC platform and course providers (Knox 2014, p.534). Furthermore, the majority of MOOCs are conducted in English and offered globally to culturally diverse students. This can be confusing or excluding for those from developing countries, particularly where interaction and dialogue are required (Liyanagunawardena et al. 2013). It is against this backdrop of MOOCs as global courses, designed in a different cultural context that the importance of seeking local methods of making sense of western-focused online courses for local Indonesian contexts can be understood. The emergence of offline communities such as IDCourserians to support online education through MOOCs might offer possibilities for addressing some of the challenges that learning online through MOOCs present, and this is what this paper aims to investigate.

This paper is organised as follows: the ‘Background’ section explains the rationale of the study and reviews recent and relevant literature associated with it. It also considers the local context for MOOCs in Indonesia in particular. The following section articulates the theories of collaborative learning and communities of practice and explains how these theoretical perspectives have been used as the theoretical frameworks of this study. The way this research was conducted is described in the ‘Methods’ section. This is followed by sections which illustrate the results of the study and the discussion which considers the relevance of these results in relation to the literature review and theory presented. Finally, the conclusions of this study are presented in the last section.

## Background

MOOCs have been evolving towards ‘global-institutional spaces’ which present themselves as both elite institutions and global classrooms (Knox 2014, p.534). In addition, MOOCs have begun to offer more standardised courses (Rodriguez 2013). This is rather different from earlier MOOCs which were mainly open courses involving a public audience in which learners participated as the co-creators of the course contents (Yeager et al. 2013). Alongside MOOC continuing popularity, which is endorsed by the mass media (Pappano 2012), some problems have begun to surface. One that appears to be gaining prominence is sustainability: for example, high dropout and low completion rates, which are reported to be below than 10 % on average (Reich 2014; Breslow et al. 2013; Belanger & Thornton 2013; Clow 2013). In response to the growing concerns over these sustainability issues, some MOOC providers have begun to campaign for the development of offline communities. Coursera, for instance, partnering with the New York Public Library, initiated a learning hub which hosts groups of people to learn particular courses (Coursera 2014). Anyone who is interested is welcome to register and invited to attend weekly meetings in the library. Sometimes, however, it does not run as expected. A personal visit (by author 1) revealed that some classes were cancelled due to no one being registered. This suggests that providing offline resources may not be enough to either initiate a learning community or to sustain MOOC learning and warrants further investigation. This is reinforced by a recent study (Veletsianos et al. 2015) that highlights the activities that learners are engaged in addition to working online in a MOOC such as social networking, note-taking, and gathering additional resources and information. However, this study was based on interviews and focused on individuals and did not suggest that any of these activities are organised around communities such as the one that we focus on in this study.

### MOOCs in developing countries

Although a MOOC is intended to bridge the education gap between developed and developing countries, there are real challenges with this. As discussed above, Knox (2014) has shown that the majority of MOOC providers are located in North America, Australia, and Europe with participants coming from across the globe to enrol in courses run by elite universities. Liyanagunawardena et al. (2013) further argue that whilst MOOCs have potential to offer education more widely and to marginalised groups in developing countries, many of these countries are still struggling with infrastructure, basic education, and limited bandwidth. This can lead to challenges for learners taking MOOCs, for example, in accessing high definition videos or managing the cultural expectations and technical challenges of engagement in synchronous chat forums. Furthermore, the ubiquity of English as the medium of instruction may disenfranchise those who are have limited or no linguistic fluency in English. Even for learners in developing countries who can manage learning in the medium of English, there may be further cultural challenges, including different norms and expectations of how to behave or interpret the behaviour of others in an online course (Liyanagunawardena et al. 2013). This suggests that more local support is needed, along the lines of the initiative of Coursera in New York but support that is more culturally sensitive to the needs of local people in developing countries.

**Fig. 1:**
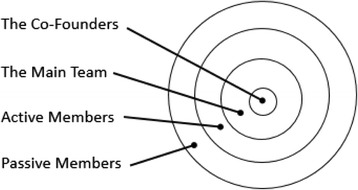
The layers of membership within the IDCourserians

In Indonesia, where provider-supported learning communities are not yet present, some MOOC learners took the initiative to form their own community, which they called *IDCourserians* and which was founded in Jakarta in April 2013 (IDCourserians n.d.). Different from the hub in New York, the community’s members organise the meetings themselves in order to decide how MOOC will be studied. Currently, from initial discussions with the co-founders of IDCourserians, it is known that the community conducts regular face-to-face meetings called ‘meetups’ and a group on Facebook. The meetups are numbered, and the most recently was the 34^th^ meetup. From these discussions as well, it seems there are different levels or layers of membership within the IDCourserians community, as can be seen in Fig. 1. The axis includes the co-founders. The next layer is the main team, who together with the co-founders, administer activities such as deciding what kind of topic will be discussed in meetups and organising the time and place. Then, the third layer is active members, who have attended the IDCourserians meetup. Finally, the outermost layer is the passive members, who only joined the Facebook group. As of 2015, there are 202 members of the IDCourserians.

### MOOC participants’ dropout behaviour

Whilst MOOC providers point to the large number of enrolments in MOOCs, there is growing evidence that the level of participation and subsequent dropout from the course is very extensive (Hollands & Tirthali 2014). Some researchers have investigated the reasons behind the exceptionally low completion and participation rates in MOOCs. Fini (2009) found that a lack of time is the most common reason why many could not finish the course. Furthermore, Fini found that the incentives the provider offers, a completion certificate, show no effect in influencing participants to complete it. Rice (2013), who joined a Coursera course, apart from finding a similar reason for not finishing (conflicting priorities and commitments due to time constraints), also found that in the discussion fora which were quiet, participants were losing track of the course, and often abandoned, preventing him from continuing engagement in the MOOC. Beaven et al. (2014) also highlight that the way MOOCs are often designed make assumptions about learners’ skills and the skills required for success in a MOOC. They further highlight the need for skills and requirements to be made more explicit to participants depending on the pedagogical approach and expectations of MOOC facilitators. Related to this, researchers have found that having insufficient prior knowledge about the subject matter can also lead to people struggling or dropping out (Belanger & Thornton 2013). They note that this condition is aggravated by the incongruity the participants perceived between what was taught and what was tested in assessment activities.

**Fig. 2:**
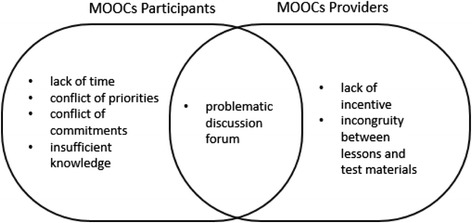
The reasons behind dropout behaviour based on their associated sources

The aforementioned reasons can be mapped into three domains: participants, providers, and their intersection as can be seen in Fig. 2. The left curve shows the problems coming from the learners, and the right side displays those associated with MOOC providers. Meanwhile, the intersection illustrates where these issues overlap.

### MOOC study groups

Although many MOOCs are designed for individual learning as a means to accommodate participants studying at their own pace (Pomerol et al. 2015), some studies show that learning MOOCs in group settings not only leads to better outcomes but also might help students to overcome obstacles such as a lack of motivation and difficulties in understanding the materials. Li et al. (2014) conducted an experiment assigning university students to watch MOOC videos together and letting them pause the videos anytime. Based on observations during a 5-week period, all groups not only paused the videos but also had discussions such as checking if they had the same understanding of the given materials. The students reported that these learning methods increased their motivation to complete the MOOC and helped them to resolve some of the challenges they faced such as language barriers and difficult concepts.

Meanwhile, Chen and Chen (2015) facilitated a MOOC learning group consisting of four university students by arranging weekly meetings in restaurants to share the participants’ experiences and progresses. The students reported that this motivated them and provided the benefits of exchanging tips to learn the course. Furthermore, Chen and Chen (2015) identify two key factors influencing the dynamics of this study group: social comparison and sense of community. Social comparison is gained from openly sharing thoughts and helping each other. Sense of community provokes individuals’ reflection on self-goals and stimulates positive competition among peers.

These studies show that there are ways in which learners may be able to overcome some of the challenges of engagement in MOOCs; however, many of these examples involve tutors setting up spaces and opportunities. What is less clear is what the potential for self-organised communities outside of formal education that support MOOCs might be. It is this topic which is the focus of this paper which investigates the IDCourserians learning community.

### Research questions

The paper draws on a qualitative study that aimed to investigate and to make sense what was actually happening in the IDCourserians as an independent MOOC learning community, in particular with respect to issues of sustainability as highlighted earlier. Accordingly, the research questions were as follows:

- What is the purpose of the IDCourserians community?
- How do the IDCourserians members learn MOOCs within their community?
- How do face-to-face and online interactions within the IDCourserians scope influence what the members do with MOOCs?

## Theoretical framework

The theoretical framework for this paper draws on two key theoretical perspectives: collaborative learning and communities of practice.

### Collaborative learning

The assumption that MOOCs are another version of collaborative learning (Gillani & Eynon 2014) suggests that to understand learning taking place in the IDCourserians context, it is important to clarify the aforementioned theory as a starting point. In a broader sense, collaborative learning refers to a situation in which two or more people, who are more or less at the same level, attempt to learn something together (Dillenbourg 1999). It takes place in a joint problem space, which is an integration of a shared goal, description of the problem, awareness of potential problem-solving action, and the relation between those three (Roschelle & Teasley 1995).

Although the term is often used interchangeably with ‘cooperative’ learning, they are, in fact, two different concepts. In cooperative learning (Johnson & Johnson 1999), an individual within the group does not necessarily have the same skill level or function. Students can split their work into several vertical divisions of labour with various objectives, where each person has a very defined role. In contrast, collaborative learning requires that the persons involved possess more or less the same skill level (Dillenbourg 1999) so their efforts can be coordinated mutually and performed together in pursuing a shared goal (Roschelle & Teasley 1995). Indeed, although there may be some division of labour between learners working together, it will tend to be at low level, horizontal (across tasks), and flexible (Dillenbourg 1999). In other words, it is a manifestation of a peer-to-peer relationship. The differences between collaboration and cooperative learning can be seen in Table 1.

**Table 1:** Comparison of collaborative and cooperative learning

Learning type	Learning goals	Learners’ knowledge	Division of labours
Collaborative learning	Shared	About the same	Low, horizontal
Cooperative learning	Individual	Mostly diverse	High, vertical

Therefore, learners who are collaborating are doing far more than working in a group and are less likely to have a defined role, roles are more likely to be flexibly, and people are more likely to work across tasks. In order to develop a sustainable, self-organised community such as IDCourserians, it could be argued that both collaborative and cooperative actions and roles are needed. This suggests that a more developed understanding of how communities are organised is required. In the next section, the theory of communities of practice is introduced.

### Communities of practice

Instead of viewing learning as a relation between students and teachers as it happens at schools, the theory of communities of practice articulates learning in terms of membership and participation within particular communities (Lave & Wenger 2005). Learning therefore is a social practice that takes place within a community. There are three elements which Wenger (1998) argues distinguishing a community of practice from other communities and are core elements that must be present. They include mutual engagement, joint enterprise, and a shared repertoire (Wenger 1998).

Mutual engagement involves participation and doing things together, including not only members’ competence but also their unique contributions, particularly when they have different roles in given communities. Such mutual engagement is not determined by geographical proximity, though in some cases, this may be helpful. For instance, in the context of a MOOC community in one city, being in the same city does not make them mutually engaged, but they are mutually engaged because they interact with each other to participate in the same activity. When this relationship is sustained, it then leads the members to negotiate their practice and the meaning of their actions.

As members interact within communities of practice, they develop a shared understanding of what binds them together. This is called as a joint enterprise (Wenger 1998), which then gives members a sense of coherence that goes beyond stated goals. Such joint enterprise is not fixed; rather, it is renegotiated by its members as the communities go on. Members develop trust between each other; hence, they become comfortable with addressing real problems and speaking openly. Different from what some might imagine as an ideal community, disagreements and tensions can fuel the process of negotiating joint enterprise and thus should be viewed as positive parts of the process.

Finally, a shared repertoire is understood to be a set of communal resources which a community produces or adopts in pursuing their joint enterprise. It may include ‘routines, words, tools, ways of doing things, stories, gestures, symbols, genres, actions or concepts’ (Wenger 1998, p.83). Such shared repertoire has its coherence within given communities. Because the repertoire of a given community is a resource for the negotiation of meaning, it is shared in an interactive and dynamic sense. Every member can utilise it, and it is through this that they develop shared meaning.

Wenger (1998) points out that knowing in a social context is considered as a manifestation of such communities of practice and this involves participating in a given practice. It is not simply acquiring knowledge by receiving information from others, as it typically happens in traditional schools between teachers and students (Sfard 2010). Therefore, in order to be competent, members must access those three aforementioned elements and remain engaged with them.

**Fig. 3:**
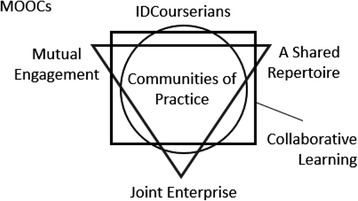
The use of communities of practice and collaborative learning as a theoretical framework to understand the IDCourserians as a MOOC community

Employing communities of practice as a theoretical framework in this study means framing what the IDCourserians members do within their community in relation to the three constituent elements. Thus, the framework as can be seen in Fig. 3 identifies which activities or entities within the community can be understood as mutual engagement, a shared repertoire, or joint enterprise. In addition, it also illuminates how this links to collaborative learning.

## Methods

This research adopted an intrinsic qualitative case study, which emphasises multiple sources of information to investigate a complex real-life situation in a bounded system perceived as the case (Stake 1995). Three data gathering methods were utilised, i.e. (1) in-depth interview (Cohen et al. 2011), (2) direct and non-participant observations (Baker 2006), and (3) document review (Mason 2002). Six interviews were conducted between 24th June and 1st July 2015 in Jakarta. Meanwhile, direct observations were carried out twice: firstly in Jakarta, on 14th July 2015 when the IDCourserians members had their 32nd meetup, and secondly via online from Bristol on 26^th^ July 2015 when the community had their 33rd meetup. In-depth semi-structured interviews were used (Cohen et al. 2011), which allow interviewers to be flexible and adaptive in terms of posing and probing with further questions in order to gain a deeper understanding of the topic. Interviews helped interpret the learning activities, roles in the community, how the community was established, relationship to Coursera, and the challenges the IDC faced. It was important to hear from active and less active members. The main language used in the interviews was Bahasa Indonesia. However, some also used English and Javanese. This was anticipated because many Indonesians speak at least two languages: the national and local languages.

The second type of observation, non-participant observation, was carried out by watching the IDCourserians members’ activities in the community Facebook group during the data gathering period, from 10th June until 6th August 2015. As explained by Baker (2006), being a non-participant means that a researcher is not present at the scene but still observes what participants do. It is commonly used to research people’s behaviour in an online environment. In the same period, the document review was also carried out by collating public documents available on the community’s Facebook group and website such as photos of previous activity, presentation slides, and weblog post documents. From the data collection, six interview transcripts, field notes, screenshots of activities, and copies of documents were gathered.

Prior to data collection, university ethical clearance was obtained, and then, an announcement containing the aims and the procedures of this research was posted in the IDCourserians Facebook group and people were given options asking if they wanted their data to be excluded and if they would participate further in the study. Following this, a detailed information sheet and consent form were sent to members who agreed to participate further.

To analyse the data, interviews were fully transcribed and field notes from observations and notes on documentary analysis were collated. The study then employed a thematic analysis approach, which is defined as ‘a method for identifying, analysing and reporting patterns (themes) within data’ (Braun & Clarke 2006, p. 79). Conducting a thematic analysis needs to follow the logic of analysing qualitative data, meaning data condensation, the process of selecting, focusing, and transforming the data; data display or organising data that permits conclusion drawing and action; and drawing conclusions and seeking verification by deciding what meaning will be presented (Ridder et al. 2014). Steps in conducting the thematic analysis following Braun and Clarke’s method were as follows:

1. Familiarisation with all prepared data by reading and rereading the interview transcripts as well as scrutinising the selected documents and screen shots.
2. Generating initial codes by highlighting meaningful sentences using different colours representing different groups of codes.
3. Searching for themes by bringing highlighted sentences with the same colour together as well as attaching them with relevant documents and screen shots.
4. Generating a thematic map of the analysis with themes and sub-themes

From this iterative process of reviewing, highlighting, selecting, and mapping collected data, forty-five themes were initially identified, which were then subsumed into three, two, and four overarching themes that illuminate the purpose of the IDCourserians, the way the community’s members learn MOOCs, and the benefit the participants perceived by joining the IDCourserians, respectively. It should be noted that these findings, generated from a qualitative case study (Stake 1995), cannot be generalised; however, they can illuminate experience and give new insights that can inform other research and practice.

The participants were recruited through a purposive sampling strategy in order to select participants with particular characteristics and to address the research questions (Punch 2009). Thus, six IDCourserians members consist of four males and two females with various degrees of engagement as can be seen in Table 2 were selected. Those are Sean, David, Dee, Ron, Sheila, and Joseph (the names are pseudonyms).

**Table 2:** A brief description of the participants

Name	Layer	Gender	Occupation	Background
Sean	Co-founders	Male	Teacher	Education
David	Co-founders	Male	Lecturer	Business
Dee	The main team	Female	Teacher	Education
Ron	The main team	Male	Employee	Pharmacy
Sheila	Heading to be passive members	Female	Employee	Finance
Joseph	Heading to be active members	Male	Employee	Economics

Both Sean and David are co-founders of the IDCourserians. Sean is a teacher in a high school in Jakarta whilst David is a lecturer in a university in the same city. Dee is Sean’s colleague. Though she was involved with the IDCourserians since the beginning, Dee considers herself as a member with moderate involvement in the main team. Meanwhile, Ron joined the IDCourserians in 2014. He is considered as one of the main team members due to his contribution to the community. On the contrary, Sheila, who joined the IDCourserians since the beginning, was considered to be a less active member because recently she was often absent from regular meetups. Joseph is a newcomer. He only attended the meetup once but was considered by community leaders to be an active member.

## Results

The findings of this research, which were thematically analysed, are organised around the three research questions as follows.

### RQ1: what is the purpose of the IDCourserians community?

#### Creating a learning place for Coursera takers

**Fig. 4:**
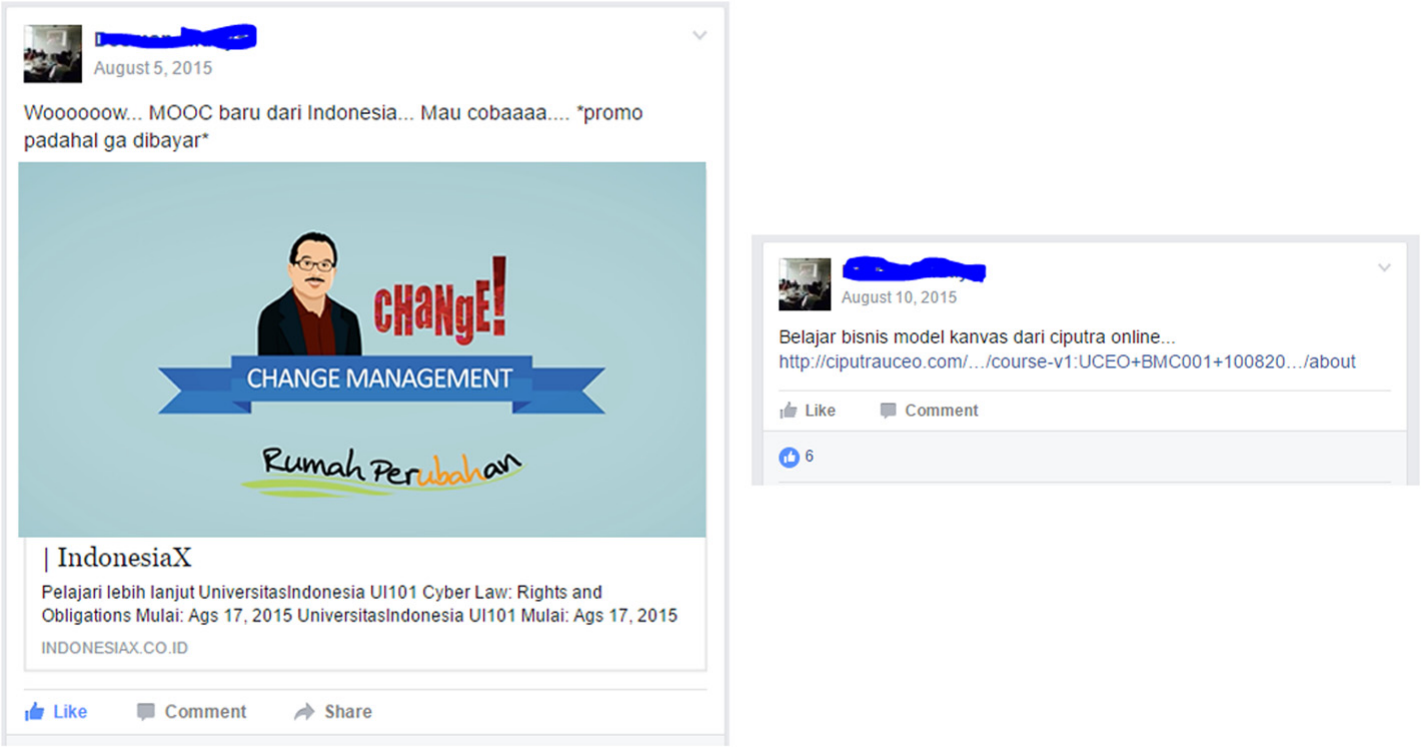
Two posts by David in the Facebook group about other MOOC providers (source: the IDCourserians Facebook group)

The co-founders of the IDCourserians reported that by forming a community, they intended to gather all Coursera takers based in Indonesia to learn together and share their experiences in joining different MOOCs. Ron explained that by doing this, the IDCourserians aim to ‘afford a networking advocate platform’. Thereby, the community allows its members to find other Coursera learners. Though the name of ‘IDCourserians’ creates an impression that the IDCourserians is designed for Coursera only, the community members interviewed clarified that it is not. The common ground of the community is the love of online learning. Hence, no matter what kind of MOOCs are chosen, all are welcomed to join. Some postings in the Facebook group also confirmed this. As can be seen in Fig. 4, the site promoted other MOOC platforms such as IndonesiaX and Ciputra UCEO.

#### Promoting Coursera

The second purpose of the IDCourserians is to promote Coursera. Members reported that during the group’s formation, they were not sure whether to focus on Coursera only or MOOCs in general. Nevertheless, because Coursera was the largest platform at the time, the community agreed to name it after Coursera. In addition, the members who had benefited by joining Coursera wanted to share their experience so that more Indonesians could also benefit.

**Fig. 5:**
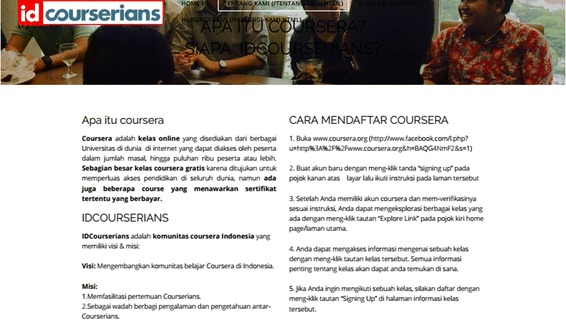
The ‘About Us’ page in the IDCourserians’ website. It gives information about Coursera (what and how to register) prior to introducing the IDCourserians community

Promoting Coursera was considered an inevitable way to develop the community since the notion of MOOCs was still in its infancy in Indonesia. Thus, it makes sense that the IDCourserians posted a brief information about Coursera and steps to participate in it prior to describing the community itself in the ‘About Us’ page on their website, as can be seen in Fig. 5.

#### Localising Coursera course content

The members reported that they found that many given examples or tasks in Coursera were not in accordance with the Indonesian context. This, according to them, made the course contents more difficult to digest. Thereby, the IDCourserians aimed to localise the content for Indonesia. Sean explained,

Doing online learning alone without interaction with local learners seems not contextual because the lessons are too Western, too US. So, we thought we need people to translate them into a local perspective. (Sean, 27 June 2015)

Related to this, there was a plan to make subtitles in Bahasa (local language) Indonesian, so that more people would get the local taste of Coursera courses. Since doing this was very time-consuming, the IDCourserians tried other efforts, one of which was hosting discussions. Ron shared his experience as follows:

When I took the course “Introduction to Communication Science”, I did not understand the examples given by the lecture. Well, one learning buddy who was more familiar with the subject, adjusted them into our culture. So he tried to connect them with similar conditions occurring in Indonesia. (Ron, 28 June 2015)

### RQ2: how do the IDCourserians members learn MOOCs within their community?

The data analysis of interviews, observation, and the Facebook group showed that the way the IDCourserians members learned MOOCs evolved as the community progressed and through its experiences of trialling new ways of learning together. The learning methods the community used can be summarised into two overarching categories: face-to-face and online learning. The first method refers to any learning condition where the members convened, called ‘meetups’, whilst the other refers to any learning condition where the members used the Internet as a medium for their learning process with other members. All methods are summarised in Table 3 and will be discussed further.

**Table 3:** The list of learning methods done by the IDCourserians members

Category(s)	Learning method(s)
Face-to-face learning	Course sharing
	Seminar model
	Semi-guided discussion
	Specific study group
Online learning	Specific online study group
	Crowd discussion
	Course review

It is worth noting that prior to deciding what kind of methods would be used to learn MOOCs together, IDCourserians main team members discussed them in community meetings. Usually, the meetings were held right after regular meetups finished. Since most of the main team of the IDCourserians were employees working in different locations, the meetings were sometimes conducted through the Internet.

#### Face-to-face learning

The analysis indicates that there were four learning methods identified under this category: course sharing, seminar, semi-guided discussion, and specific study groups. These names came from the participants. As mentioned earlier, all these methods of learning mostly involve discussion, albeit in different formats:

Doing an online course is like reading a book. We then need a medium for discussions. In learning, we cannot just listen and receive, take it for granted. We have to share, discuss, hone, and apply it. I think Coursera just limits itself toward essays or quizzes. There has not really been discussion yet. That is what is missing from the learning element which should have existed in the discussion forum … We need to meet face to face, to share, and to discuss. (Sean, 27 June 2015)

**Fig. 6:**
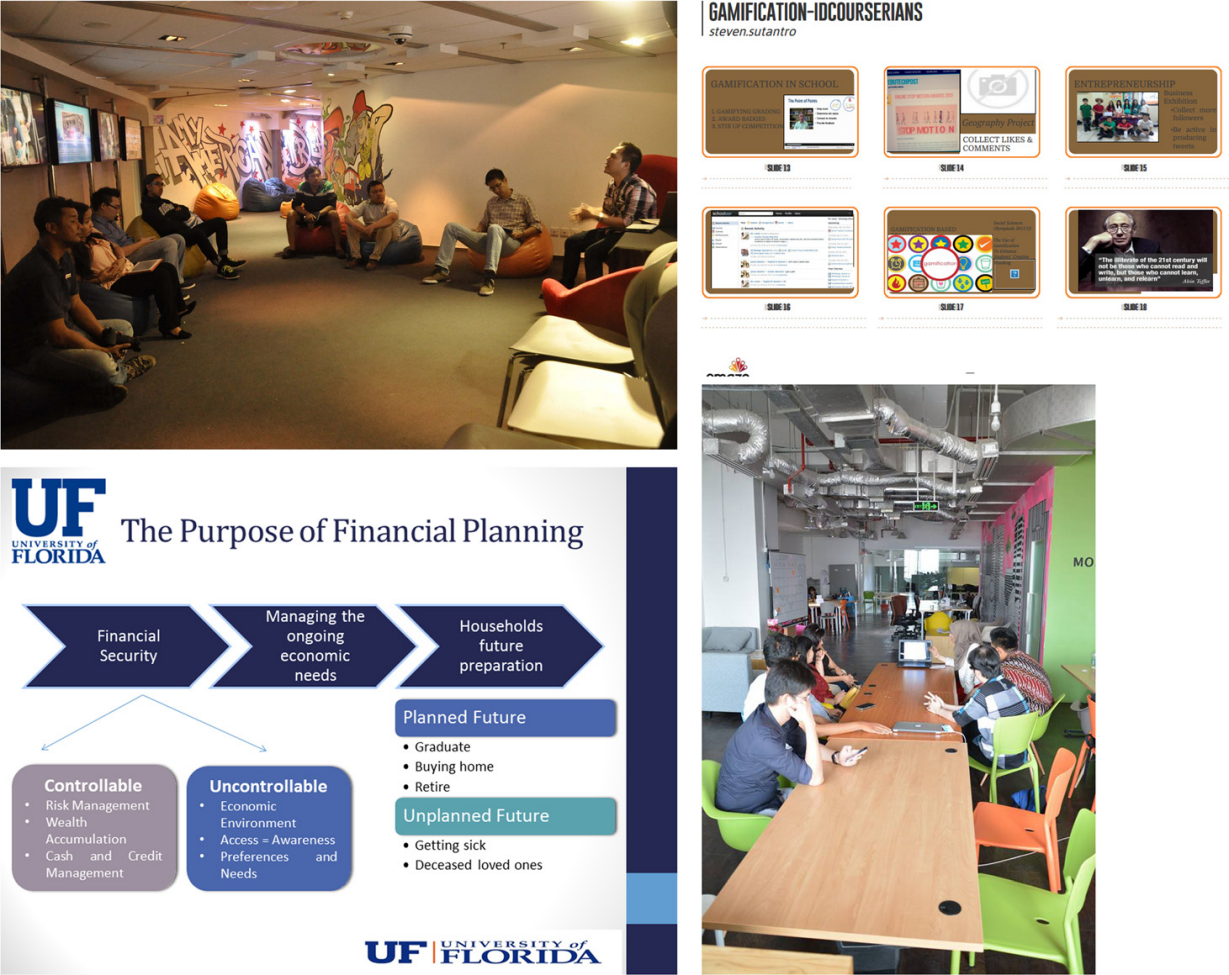
Some examples of the course sharing model in a group. From the *upper left clock-wise*: (1) Sean shared Gamification, (2) Sean’s slides on Gamification, (3) Sheila’s slide on financial planning and (4) Sheila shared the financial planning course (source: the IDCourserians Facebook group)

The first learning method identified was *course sharing* (Fig. 6), which refers to a meetup in which a member voluntarily presented the course they joined and facilitated the discussion process following the presentation. As previously mentioned, it did not require that the members were taking the same course. This format only happened three times in borrowed private spaces in Jakarta. However, as of 2015, course sharing was no longer used. Thereby, the IDC decided to move to another learning method.

**Fig. 7:**
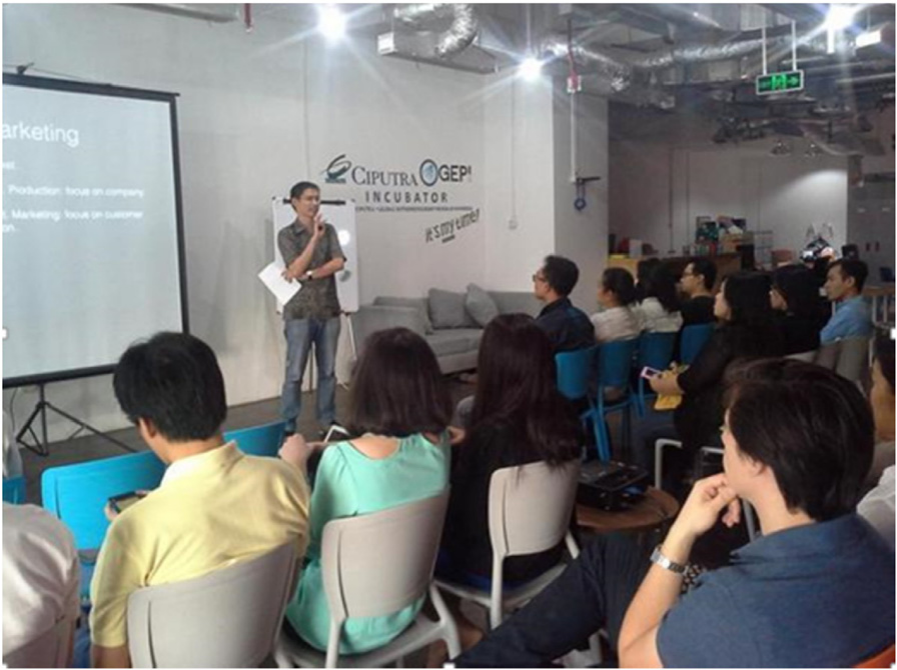
Example of seminar model

The next learning method was *seminars* (Fig. 7). This method shared characteristics with course sharing but had two distinctive features. Firstly, the attendance was broader as most were considered ‘public’. Secondly, it was one-directional sharing as opposed to being interactive. The seminar format was carried out twice in a hall belonging to another organisation, which lent it to the IDCourserians for free. As of 2015, this method was also no longer used, because not all members were interested in the given topics, it was therefore too formal a method of supporting community members.

**Fig. 8:**
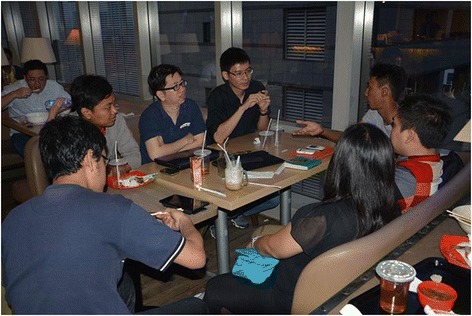
Example of semi-guided discussion. How the discussion is run (source: the IDCourserians Facebook group)

Another type of learning that has since emerged is *semi-guided discussion*. This method encourages those attending to discuss their thoughts and experiences of joining Coursera courses. In this model, one or two persons lead the process. Since each member has their own interests, the IDCourserians aim to choose a topic as general as possible, for instance, ‘online learning in Indonesia’. Semi-guided discussions usually take place in cafés in central Jakarta as seen in Fig. 8. Although this learning method was still being used at the time this study was conducted (2015), several problems surfaced during interviews and in meetings observed. Firstly, sometimes the topic was found to be too general, which made the discussions less focused. Based on the direct observations, the discussion went off-topic several times, and in the middle, it broke up to several sub-forums. Secondly, only a few members were able to join this meetup, which made the discussion a bit limited.

**Fig. 9:**
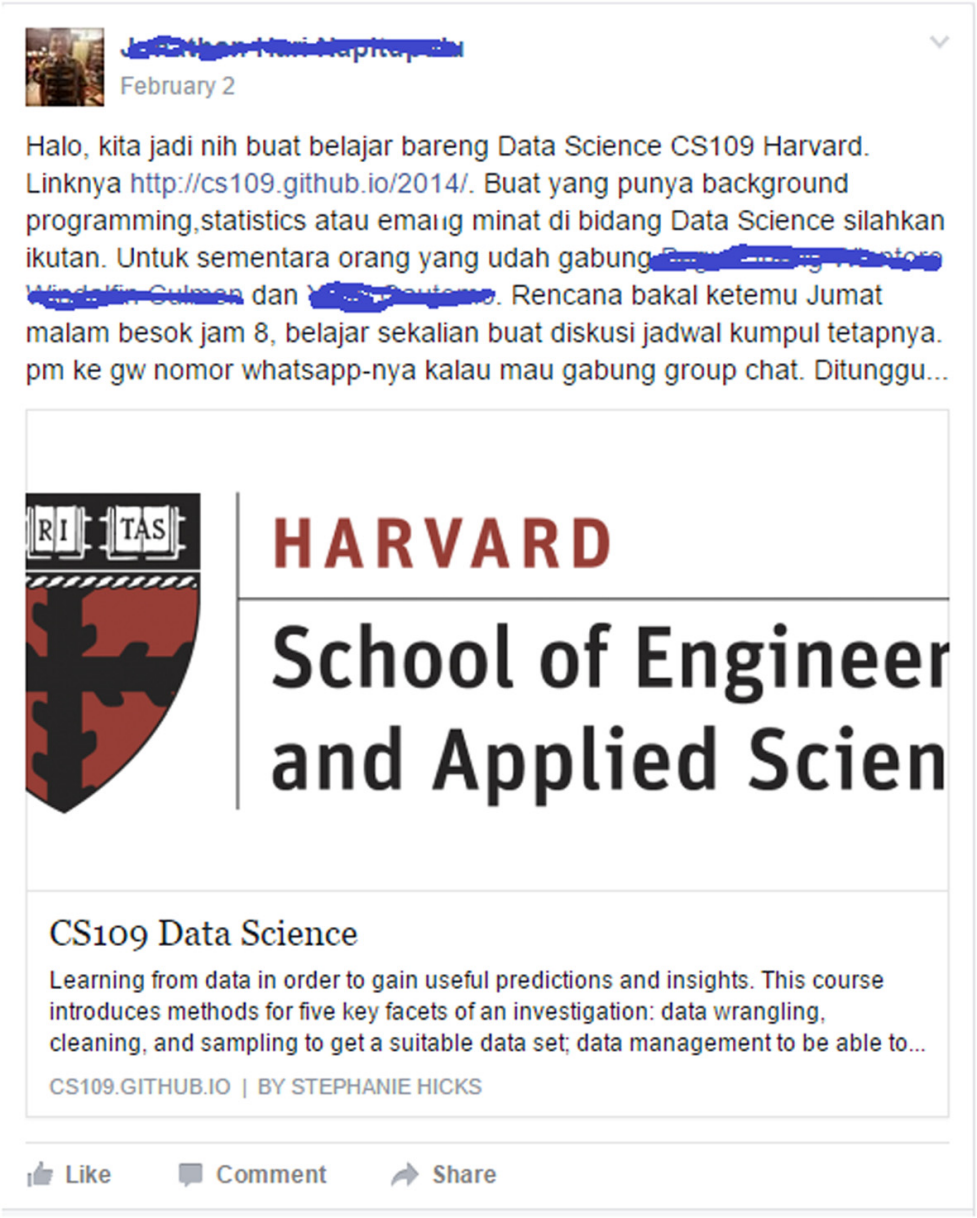
One example of an open invitation to a specific study group posted in the Facebook group (source: The IDCourserians’ Facebook group)

Specific study groups were different from the three aforementioned learning models under the face-to-face category, as they usually began with a member posting an open invitation to study certain courses together in the community’s Facebook group. This is seen in Fig. 9. Should others studying the same courses could then reply and agree to the proposed time, they would arrange their own meetup.

It is argued that this counts as one of the learning methods because it uses the community as a channel. In this respect, it reflects the IDCourserians’ aim to afford a networking platform. One interviewed participant who had done this was Ron. He and other members learned an operation management course in a co-working space in Jakarta.

When it comes to learning a specific course, we usually make person-to-person appointments. We, I and several members, enrolled in an operation management course from Warton … then we sat up meetings…. (Ron, 28 June 2015)

#### Online learning sessions

As well as face-to-face learning methods, IDCourserians members reported that they used the Internet as a medium to learn together. There are three learning types that can be subsumed under this category: online specific study groups, crowd discussions, and course review. The first type is a synchronous form where the members participate at the same time. Crowd discussions and course reviews were asynchronous, where learners participated in the discussions at different times.

An online specific study group is an online version of a face-to-face-specific study group. This was started because of barriers the group faced when conducting the offline version such as Jakarta’s traffic jams which made meeting up more challenging.

**Fig. 10:**
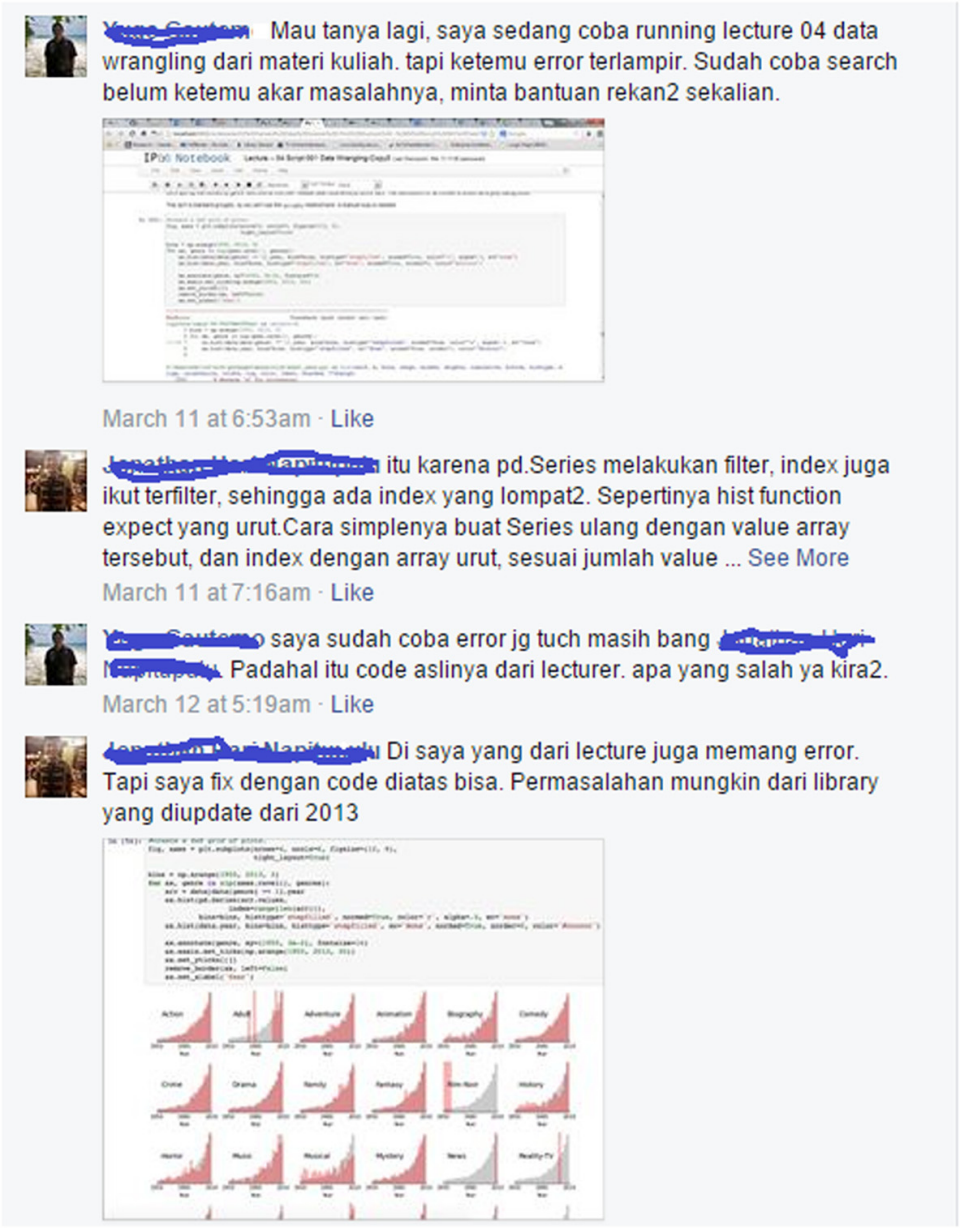
Example of a crowd discussion. A member posted their problem and others tried to help to solve it

*Crowd discussions* consisted of loose ‘conversations’ that took place in the Facebook group. The topic and the time were not predetermined as any member could participate at any time. As can be seen in Fig. 10, a member experienced difficulty with his course on computer science. He posted the difficulties by using a screenshot and got replies and solutions from other members. The fact that there are more than 200 members within the Facebook group makes the crowd discussion one of the easiest ways to get information related to MOOCs. However, sometimes, members reported that there was a long wait time for answers.

**Fig. 11:**
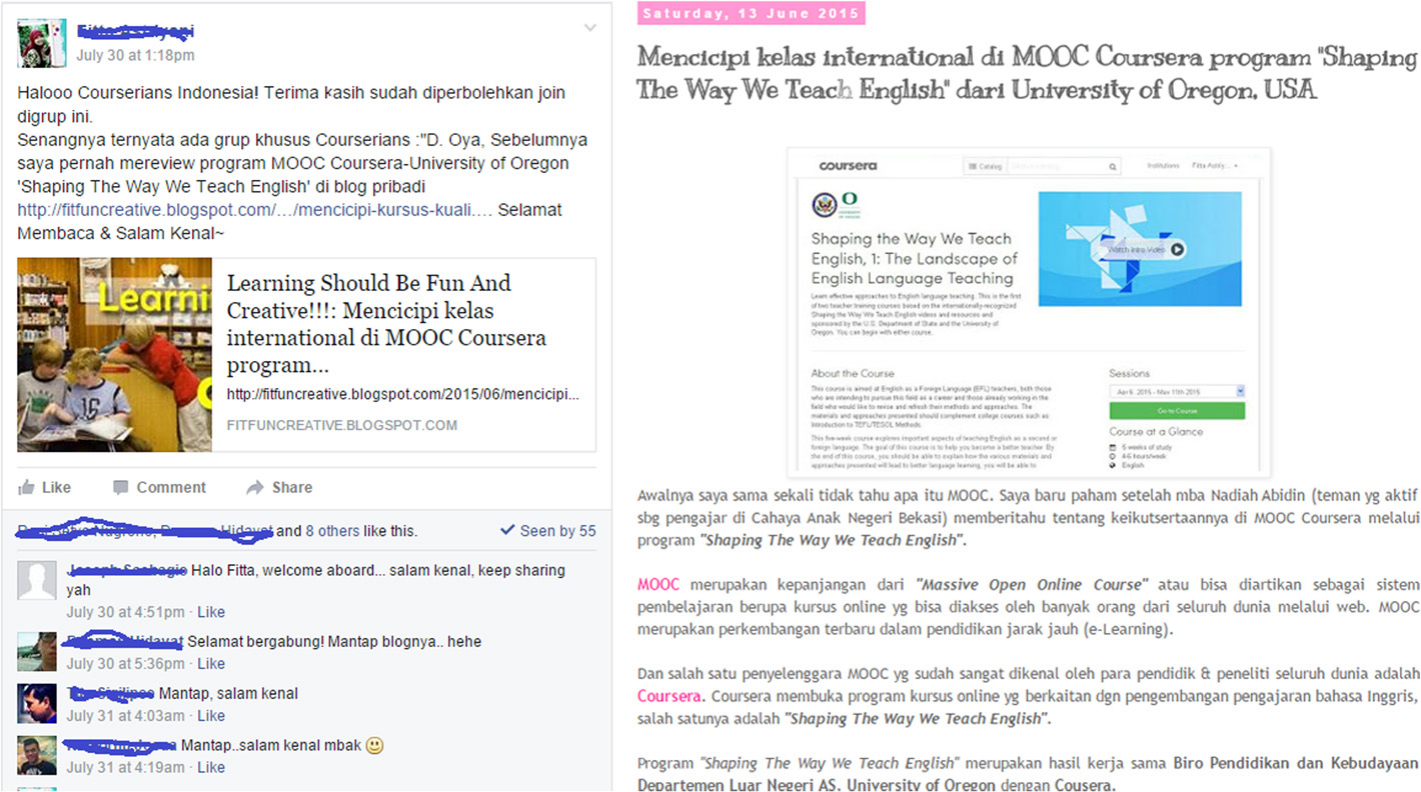
Example of course review. *Left*: A member posted their review on their personal blog in the Facebook group. *Right*: The review of a Coursera course about teaching English

Another type of learning method which was *course review* shares some characteristics with course sharing, but a blog is used as the medium instead. In this learning format, members review courses they take and provide testimonies about them to others. Usually, they write these in personal blogs and/or in the community weblog. Links to personal blogs or the weblog are shared in the Facebook group as can be seen in Fig. 11 and through the community’s Twitter accounts.

### RQ3: how do face-to-face and online interactions within the IDCourserians scope influence what the members do with MOOCs?

#### Increasing motivation to learn MOOCs

By joining the IDCourserians and getting involved in its activities, the members reported that they were more motivated to learn new courses and to finish what they started. This is because they feel that they receive inspiration from each other. Furthermore, some of them also posted their achievements in the Facebook group, which supports others to reflect on their own progress. Some also used the platform to remind each other about courses assignments.

However, for some members, motivation to keep learning did not necessarily mean having to accomplish MOOCs and get another certificate. Watching all available videos without doing the quizzes was considered more than enough to learn something new. The important thing was they acquired knowledge in their fields of interest.

Because I do not pursue the certificate, I do not pursue anything but knowledge. Let say my goal is knowledge only. (Dee, 26 June 2015)

#### Enhancing information around MOOCs

Interactions within the IDCourserians community suggest that this enhances what the participants know about Coursera and its courses. Furthermore, members’ testimonies help make shared information unique, relevant, and more contextual.

There were some members who had taken that course and they explained several things. Yes, a, b, c concepts. Those would be my expectations should I take that course as well. (Joseph, 29 June 2015)

Sharing appears to help members determine the courses that are right for them. Sometimes, they are doubtful about a course, but later, they realise its benefit from what others share.

There might be a course I did not know was good…marketing for example. At first, I thought it was useless. But there is other member joining that course, asked me to join and telling me what they get. I think it is interesting, then I decide to join it. (Sean, 27 June 2015)

#### Helping to overcome difficulties in learning MOOCs

By joining meetups, members were also able to share their learning difficulties. Although there is no tutor, typically, there would be someone who had mastered more in that course and they could help others to master the subject, as this quote shows:

I clearly remember when I took inferential statistics, I was given a data set, Google report, for a year. I was lost about what I should do with that abundance of data … They taught me how to deal with it. So learning together is really helpful. (Coursera) delivers course materials the way they do in their countries. We here in Indonesia do not like that. Meetups, learning together with other members help the (mastering) process. (Ron, 28 June 2015)

Furthermore, participating in meetups helps to regulate learning processes. In this respect, friends accompany each other in learning MOOCs.

I thought my barrier was English, but when I took a course in Ciputra, I still did not finish. So, after I researched it … I just came to realise that my learning style is getting together with others and doing discussions with them, not doing it individually. (Dee, 26 June 2015)

#### Fulfilling social needs

The participants agreed that they preferred not to learn MOOCs alone. They wanted to have friends or ‘classmates’, not just strangers who take the same courses. By joining this community, they were able to meet others who share the same passions and this appears to fulfil their social needs and sense of community and belonging:

I got a sense of togetherness, I’m not alone. (I was) happy because there were others who take (the same MOOCs) … It was difficult to find a learning community. Online learning was more about the brain thing. It seemed that we are very ‘geek’, with online learning. There weren’t many people doing this… So, when I found them, I was happy there would be people I could share with. (Sheila, 24 June 2015)

As Wenger (1998) explains, communities of practice are about members sharing a common ground. This is clearly reflected in the quote above. Members of the IDCourserians were happy to realise that there were others doing the same thing. Once they realised this, they started to negotiate their joint enterprise. A quote from Sean reflects how this happens.

I have a place in which I can share what I know … I could be connected with fellows who also learn Coursera, get support, and are able to share … At least, should I talk about Coursera, there are those who can support (me). (Sean, 27 June 2015)

## Discussion

**Fig. 12:**
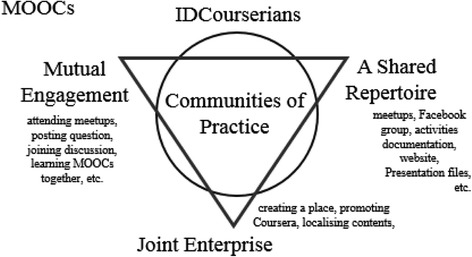
The elements of practice in the IDCourserians

As shown in the theoretical framework in Fig. 3, the IDCourserians community can be interpreted using the dual lenses of communities of practice (Lave & Wenger 2005; Wenger 1998) and collaborative learning (Roschelle & Teasley 1995;Dillenbourg 1999). Adopting this dual framing allows us to understand the ways in which participants have come together and why and how their meaning making and shared practices (including the learning activities that were established) have developed and the purpose and sustainability of the community as a mechanism for supporting learning in online courses and shifting the emphasis from independent activities in a MOOC to a joint activity. We have therefore interrogated the thematic data using the key concepts from these two theoretical perspectives (see Figs. 3 and 12). In particular, the three dimensions of communities (mutual engagement, joint enterprise, and shared repertoire) and the features of collaboration and co-operation in learning activities (synchronicity, shared goals, joint problem space, relationship with peers, division of labour) have been employed to further explain and interpret the data and outcomes of the thematic analysis (Mason 2002). In the following sections, we discuss these concepts in relation to the findings and relevant recent literature.

### The IDCourserians as a community of practice

What the IDCourserians members do together face-to-face and online can be considered as a form of mutual engagement (Wenger 1998). In doing so, each member has unique contributions. For instance, there were persons becoming presenters in semi-guided discussion, whilst others preferred using the weblog as a medium for sharing. The IDCourserians developed a joint enterprise by expanding their shared goal from learning MOOCs to promoting Coursera and localising its contents. The members appear to be coherently heading in the same direction. Out of this, a sense of community emerges. The enterprise having developed without intervention from the provider resonates with what Wenger (1998) points out, that such joint enterprises are negotiated by its members, not by outside entities. The way IDCourserians arranged meetups, produce weblog posts, and shared information on Facebook can be considered as a shared repertoire. These are unique methods that reflected the personality of the community. As Wenger (1998) argues, such repertoire is shared in dynamic and interactive ways, as seen in the IDCourserians. For example, in the Facebook group, any member could use it to ask and respond to questions.

Drawing the findings presented earlier, it can be argued that the IDCourserians are a community of practice (see Fig. 12). This contradicts the previous studies done by Can (2013) as well as Gillani and Eynon (2014) who argue that groups of MOOC learners learning online are far from the definition of communities of practice. It seems that the method and intensity of interactions are vital. In the above studies, the learners’ interactions were limited to the Internet and discussion forums only. In the present study, the members interact more frequently both face-to-face and online. Since mutual engagement is the thing that defines community (Wenger 1998), it makes sense why Can (2013) and Gillani and Eynon (2014) come to their conclusions. In this respect, the participants in their studies seemed to have less time to do things together, which made them less mutually engaged, whereas in the study reported here, members came together specifically work together on a joint enterprise which was more about making MOOCs work at a local community level than working on a specific MOOC.

### Collaborative learning within the IDCourserians

Though all learning methods developed in the IDCourserians take the form of study groups, it is argued that only face-to-face- and online-specific study groups can be considered as collaborative learning (Dillenbourg 1999). Meanwhile, other methods such as course sharing, seminars, and crowd discussions could be considered as cooperative learning (Johnson & Johnson 1999). The learner-to-learner interaction reflects a vertical division of labour, where one acts as a coach. According to Dillenbourg (1999), this is a feature that distinguishes cooperative from collaborative learning.

However, it seems that in practice, cooperation and collaborative learning were more entangled in the IDC community. As an illustration, Sean and Dee were educators and following education courses. Sean delivered gamification in course-sharing, which involved Dee and other audiences with no background in education. On the one hand, both Sean and Dee still engaged in collaborative learning since, as Roschelle and Teasley (1995) argue, they studied education and are able to maintain the joint problem space. But when the point of view is expanded to the group, Sean and Dee become coaches for other members. Thereby, it is argued that collaborative learning in the IDCourserians context is a matter of degree, ranging from learning from full collaboration to learning with less collaboration

Furthermore, the fact that the community prefers face-to-face study groups benefits the members in increasing the intensity level of collaboration. Doing so also increases the level of synchronicity (Dillenbourg 1999), which enables the members to give each other instant feedback. Thereby, the learning process becomes more livelier. On the contrary, discussions taking place over Facebook or blogs, and although they also afford collaborative learning, they display less intensity of collaboration than the face-to-face study groups. For example, as shown in the findings, some questions in some posts were responded to after a very long time.

### Benefits of joining the IDCourserians

**Fig. 13:**
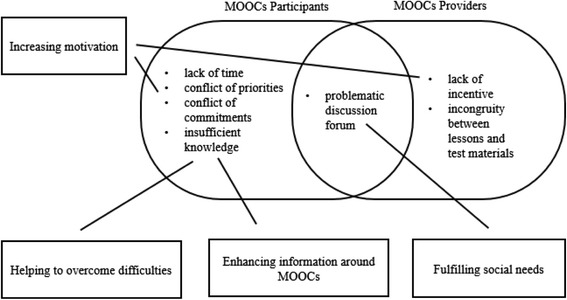
The benefits of joining the IDCourserians are the opposite of the reasons of leaving MOOCs

The benefits of joining IDCourserians reported by participants echo previous findings that learning MOOCs together leads to better outcomes (Chen & Chen 2015; Li et al. 2014). In contrast, they are the opposite of the reasons behind participants’ dropout behaviour as can be seen in Fig. 13. In Fini’s (2009) study, the participants did not find the completion certificate worth being pursued. Conversely, in the IDCourserians’ case, knowing others receive certificates led the members to be motivated to get one also, because of the peer-to-peer relationships they have built up. Meanwhile, the way the model of IDCourserians helps in enhancing information around MOOCs and in overcoming difficulties counteracts the problem of insufficient knowledge (Belanger & Thornton 2013). Listening to others’ testimonies also appears to give participants an image of how the course would look and what should be done for preparation. These are things that the learners in Belanger and Thornton’s (2013) study do not get until they joined the course. Therefore, they left as if it did not meet their expectations. Furthermore, the mode of IDCourserians that assists in fulfilling social needs suggests the opposite of feeling abandoned, which according to Rice (2013), makes the discussion forums in MOOCs problematic. The findings in this study appear to show that face-to-face discussions are more beneficial that strictly using online forums and suggest that forming a community of practice like IDCourserians might offer a solution to (some) sustainability issues. This practice benefits both participants and providers of MOOCs, since problems are mitigated from both sides.

These findings also suggest that more engaged members receive more benefits and compliment Wenger’s (1998) argument that in order to be competent members of communities of practice, participants must find ways to access its three constituent elements: mutual engagement, joint enterprise, and shared repertoire.

## Study limitations

It should be noted, since the methodology of this study is qualitative and it is based on a single case study, the findings are therefore highly contextualised and not directly generalisable. The study took place in Jakarta with a particular group of people, and it may be that in different contexts or cultures, very different outcomes would have been found. Furthermore, the data collection took place over 2 months (June and July 2014) which was a fairly short timescale to consider the development of this group. The timing was limited by the availability of both the researcher and the participants. Nevertheless, case studies showing detailed experiences and investigating historical perspectives on development can shed light on wider and longer term issues and can also suggest areas for further research and improving practice. There will be many similar contexts, particularly in other developing countries, and therefore, this study can have wider relevance. Another challenge this study faced was the researcher and the participants were in different locations. Had they been in the same location continually throughout the period of the study, the interaction with the groups and observations of their activities could have been more longitudinal and intensive which would have helped the understanding of the problem being investigated.

## Conclusions

This study investigated what was actually happening in the IDCourserians as a MOOC learning community. A qualitative case study with three methods of data gathering was combined with a thematic analysis approach to answer the four research questions as follows:

- The purpose of the IDCourserians community is to create and sustain a collaborative, community-based support for Coursera takers, to promote the use of it in Indonesia, and to localise Coursera course contents.
- The IDCourserians members learn MOOCs through face-to-face learning (course-sharing, seminars, semi-guided discussions, and specific study groups) and online learning sessions (specific online study groups, crowd discussion, course reviews).
- Face-to-face and online interactions within IDCourserians benefit participants by increasing their motivation to learn MOOCs, enhancing information around MOOCs, helping to overcome difficulties in learning MOOCs, and fulfilling social needs.

The IDCourserians have developed their community as an attempt to address the drawbacks of global MOOC practices. Whilst the providers have tried to address similar issues through delivering discussion forums, providing teaching assistants, and forming learning hubs in some places, these are very different to the IDCourserians model which is community led. Furthermore, in Indonesia as in other developing countries, where English is not the main spoken language and the hub model is not yet provided, a more local response is needed. Creating a member-led face-to-face community in which the members know each other, have more intense interactions, and share the same goals is a possible solution to these challenges. This shows that online learning platforms may require face-to-face interactions and similar community-based hubs for successful completion of courses.

The benefits the members received by joining the IDCourserians seem to have resonated with some of the pedagogical and cultural problems associated with global MOOCs provided outside of local communities. Therefore, MOOC providers and educational institutions should assist in creating face-to-face communities in local contexts to help learners engage with and improve their interactions with the global MOOC courses, as this may be a better way for learners to experience MOOCs and make their learning more meaningful. This is also a potential means of overcoming the common difficulties in MOOCs, such as a lack of motivation and difficulties in interpreting the material through developing collaborative support activities within a community of practice, and even better, would be for the global institutions to consider a more distributed strategy to encourage more online course provision in developing countries in local languages designed to meet the needs of local people and with support hubs along the lines of IDCourserians. Future research might investigate the benefits of participation in learning hubs founded by MOOC providers. It would be interesting to examine how these hubs impact on students’ experiences, engagement, and outcomes compared with self-organising communities such as the IDCourserians community reported in here.
